# Age-related considerations in the relationship between educational level and metabolic syndrome prevalence

**DOI:** 10.34172/jcvtr.33084

**Published:** 2024-03-13

**Authors:** Fatemeh Omidi

**Affiliations:** Shahid Beheshti University of Medical Sciences, Tehran, Iran

## Dear Editor,

 I am writing to discuss an intriguing aspect of the study “Prevalence of Metabolic Syndrome in East Azerbaijan-Iran and Its Determinants Factors” by Ali Farshbaf Khalili et al., as published in J Cardiovasc Thorac Res.^1^ The study’s findings on the association of lower educational levels with higher prevalence of metabolic syndrome (MetS) and the increased prevalence of MetS in older age groups warrant a deeper analysis, particularly considering the potential confounding effect of age.

 The study astutely identifies lower educational attainment as a factor associated with higher MetS prevalence. Concurrently, it notes an increased prevalence of MetS in older individuals. This raises a crucial question: could the observed correlation between lower education levels and higher MetS prevalence be partly attributed to the age factor? Older individuals are more likely to have had less access to education historically, which could mean that the higher prevalence of MetS in this group is not solely due to educational level but is also influenced by their older age.

 To accurately discern the independent effect of educational level on MetS prevalence, it is essential to employ analytical methods that account for the confounding impact of age. Such analysis would provide a clearer understanding of whether the relationship between education and MetS prevalence is direct or is significantly influenced by the higher age of the lower-educated population.

 This consideration is vital for devising effective public health strategies. If the relationship between lower educational levels and higher MetS prevalence is largely influenced by age, interventions might need to be more age-specific, addressing the unique needs of older populations. Alternatively, if education independently affects MetS risk, broader educational and health literacy initiatives would be required.

 In conclusion, the study by Ali Farshbaf Khalili et al. presents important findings on the epidemiology of MetS in East Azerbaijan. However, further analysis considering the confounding effect of age on the relationship between educational level and MetS prevalence is essential to develop targeted and effective public health interventions.

## Competing Interests

 The author declares no conflict of interest in this study.

## Ethical Approval

 Not applicable.
